# 4243 Developing clinical research units to improve quality, efficiency, and cost effectiveness within an academic institution

**DOI:** 10.1017/cts.2020.207

**Published:** 2020-07-29

**Authors:** Peg Tsao, Ashley Dunn, Kenneth W Mahaffey

**Affiliations:** 1 Stanford University School of Medicine

## Abstract

OBJECTIVES/GOALS: The Stanford CTSA Program has started to create Clinical Research Units (CRUs) with the goal to establish CRUs in all clinical departments by the end of 2020. CRUs will be responsible for managing the portfolio of projects proposed and conducted by faculty within departments. CRUs will be responsible for reviewing all clinical research studies. METHODS/STUDY POPULATION: CRUs will be an integral part of the Stanford’s research infrastructure, tasked with 5 key functions to ensure clinical research conducted by Stanford investigators: scientific merit, feasibility, funding, compliance, progress. Each CRU will review all clinical research projects proposed by investigators within the department prior to moving forward with IRB review. Studies will be evaluated annually to ensure compliance with the protocol, applicable laws and regulations, and recruitment goals. The Stanford CTSA will provide guidelines, SOPs and personnel to assist CRUs. In fall 2019, a landscape analysis of SoM clinical departments was conducted to identify:

1. similar existing CRU-like systems,
2. unique needs of departments/divisions for developing CRUs and
3. barriers to implementation.

RESULTS/ANTICIPATED RESULTS: Challenges the pilot CRU has faced include communication and concerns regarding additional obstacles to conducting research. However, as study teams moved through the initial CRU formation, the feedback was overwhelmingly positive. Study teams were appreciative of the constructive feedback and the support for setting up studies. Results from the landscape analysis identified CRU-like systems in 5 departments and highlighted concerns regarding resources needed to implement CRUs. Based on feedback from the landscape analysis, a faculty and operational lead was identified in each clinical department to oversee CRU implementation. Facilitated by CTSA personnel, CRU leads have met quarterly since April 2019. Meetings consist of discussing expectations, sharing ideas and identifying potential roadblocks. DISCUSSION/SIGNIFICANCE OF IMPACT: CRUs will constitute a new organizational structure that consists of teams of investigators and staff to promote high quality, efficient clinical research and enhance collaborative opportunities. The CRU leadership will champion new initiatives in CTR and create pathways for investigators to access research infrastructure and resources. CONFLICT OF INTEREST DESCRIPTION: NA.

